# Electrophysiological Assessment of Newly Synthesized 2,3-Benzodiazepine Derivatives for Inhibiting the AMPA Receptor Channel

**DOI:** 10.3390/molecules28166067

**Published:** 2023-08-15

**Authors:** Mohammad Qneibi, Hanan Jumaa, Sosana Bdir, Nawaf Al-Maharik

**Affiliations:** 1 Department of Biomedical Sciences, Faculty of Medicine and Health Sciences, An-Najah National University, Nablus P.O. Box 7, Palestine; s12027767@stu.najah.edu; 2 Department of Chemistry, Faculty of Sciences, An-Najah National University, Nablus P.O. Box 7, Palestine; hjumaa97@gmail.com

**Keywords:** 2,3-benzodiazepine, AMPA receptor, GluA2, amino group, desensitization, antagonist

## Abstract

Three major subtypes of ionotropic receptors regulate glutamatergic synaptic transmission, one of which is α-amino-3-hydroxy-5-methyl-4-isoxazole propionic acid (AMPA) receptors (AMPARs). They are tetrameric, cation-permeable ionotropic glutamate receptors found across the brain. Abnormalities in AMPA receptor trafficking and synaptic assembly are linked to cognitive decline and neurological diseases such as Alzheimer’s, Parkinson’s, and Huntington’s. The present study will investigate the effects of four novel 2,3-benzodiazepine derivatives on AMPA receptor subunits by comparing their effects on synaptic responses, desensitization, and deactivation rate in human embryonic kidney cells (HEK293T) recombinant AMPAR subunits using whole-cell patch-clamp electrophysiology. All four 2,3-BDZ compounds showed inhibitory activity against all the homomeric and heteromeric subunits tested. While the desensitization and deactivation rates in 2,3-BDZ-1 and 2,3-BDZ-2 decreased and increased, respectively, in the other two compounds (i.e., 2,3-BDZ-3 and 2,3-BDZ-4), there was no change in the desensitization or deactivation rates. These results contribute to a better understanding of AMPARs by identifying potential 2,3-BDZ drugs that demonstrate inhibitory effects on the AMPAR subunits.

## 1. Introduction

The central nervous system (CNS) is regarded as the processing center of the human body and the most vital part of the human body as a whole [1]. Besides being responsible for learning and memory, the CNS facilitates ongoing repairs to sustain active function and regulate the body’s physiological processes [2]. The balance between excitatory and inhibitory neurotransmitters is crucial in various physiological processes. GABA (γ-aminobutyric acid) and glutamate are essential to maintaining this balance. Gamma-aminobutyric acid (GABA) functions as the principal inhibitory neurotransmitter inside the central nervous system (CNS), counteracting the excitatory actions exerted by glutamate, which serves as the major excitatory neurotransmitter. Collectively, these mechanisms contribute to regulating neuronal activity and managing synaptic transmission, promoting optimal functioning of the central nervous system (CNS) by preventing excessive excitement that might potentially result in developing neurological diseases. A comprehensive grasp of the underlying functional association between GABA and Glutamate is crucial in neuropharmacology, particularly concerning drugs such as benzodiazepines and 2,3-benzodiazepines (2,3-BDZ), which exert their effects via modulating these neurotransmitters [3].

The brain is subjected to several systems that control and maintain many activities as a delicate organ. However, the exact mechanisms that protect it from defects may also produce disorders due to impaired functions, resulting in various neurological conditions. As a complex and vulnerable organ, the brain depends on several defensive systems to sustain its optimum functioning and safeguard itself against possible damage. The protective mechanisms include the blood-brain barrier, immunological reactions, and the brain’s capacity for neural plasticity. Although essential for maintaining brain health, these systems may lead to the development of neurological diseases when they malfunction. The blood-brain barrier is a protective mechanism that restricts the entry of detrimental chemicals into the brain. However, this physiological barrier may impede the efficient administration of drugs intended to address neurological disorders. The immune system, which is intricately constructed to safeguard the body against infectious agents, has the potential to malfunction and result in autoimmune illnesses that specifically affect the brain. Neural plasticity, an essential mechanism for acquiring knowledge and adjusting to new circumstances, may lead to maladaptive alterations contributing to chronic pain and mobility difficulties.

Furthermore, abnormalities in neurotransmitters and the influence of hereditary factors have been identified as potential contributors to neurological disorders, including Parkinson’s disease, Alzheimer’s disease, and epilepsy. Neurodevelopmental diseases such as autism spectrum disorder may arise due to abnormalities during crucial developmental periods. In addition, oxidative stress and excitotoxicity have been implicated in the pathogenesis of neurodegenerative disorders such as Alzheimer’s disease and traumatic brain trauma. Gaining a comprehensive understanding of these systems and their possible dysfunctions is crucial to progress in developing therapies for neurological illnesses and safeguarding the overall health of the brain [4,5,6]. Neurodegenerative diseases are marked by the slow loss of groups of sensitive neurons to certain stimuli. This differs from the loss of specific neurons caused by physiological or damaging disorders. Neuron-related diseases include neurodegenerative diseases such as Alzheimer’s, Parkinson’s, and Huntington’s, as well as neuro-traumatic diseases such as stroke, traumatic brain injury, spinal cord injury, and neuropsychiatric problems such as depression, autism, and hyperactivity disorders [5]. Due to the complexity of the CNS, several factors are connected with the etiology of neurological disorders [6]. Almost every neurological condition affects synapse function in the brain, either directly or indirectly. However, each condition affects the neuronal synapses in distinct ways, impairing synaptic transmission and/or plasticity [7].

The majority of synapses present in the nervous system are classified as chemical synapses, wherein the release of a neurotransmitter from the axon of the presynaptic neuron facilitates the process of synaptic transmission. This neurotransmitter subsequently diffuses across a narrow synaptic cleft and interacts with receptors located on the dendrites of the postsynaptic neuron [8]. Glutamatergic neurotransmission underlies most of the fast synaptic neurotransmission in the CNS [8]. Glutamatergic synaptic transmission is regulated by three primary subtypes of ionotropic receptors, one of which is the α-amino-3-hydroxy-5-methyl-4-isoxazole propionic acid (AMPA) receptor (AMPAR) [9]. AMPARs are tetrameric, cation-permeable ionotropic glutamate receptors found across the brain. The number of AMPARs in a given synapse ranges from tens to hundreds, corresponding with synaptic strength in mature synapses. The efficacy of synaptic connections between nerve cells can be strengthened or deteriorated depending on the number of AMPA receptors at the synapse [10,11,12]. It has been established that abnormalities in AMPA receptor trafficking and synaptic assembly processes are associated with cognitive decline and various neurological disorders [13,14].

Functional AMPA receptors are homo- or hetero-oligomeric assemblies comprising combinations of four subunits, GluA1, GluA2, GluA3, and GluA4 [15]. The GluA2 subunit confers calcium impermeability to natural AMPA receptor channels. The scarcity of GluA2-containing AMPA receptors inside motor nerve cells results in heightened calcium ion permeability, elevating susceptibility to excitotoxicity after AMPAR activation. The GluA2 subunit is subjected to an RNA editing mechanism that converts glutamine to arginine. While most AMPA receptors in the brain are Ca^2+^-impermeable, they have an edited GluA2 subunit [16,17]. Each subunit has a large extracellular amino-terminal domain (ATD) that aids with the receptors’ assembly, trafficking, and modulation. It also has a ligand-binding domain (LBD) formed by two segments, S1 and S2, that are important for binding and activation gating, a transmembrane domain (TMD) that forms the membrane-spanning ion channel, and a cytoplasmic carboxy-terminal domain that helps with receptor localization and regulation [18,19,20]. The ATD, LBD, and TMD impact the tetrameric assembly of AMPARs [21]. Each subunit comprises three transmembrane helices (M1, M3, and M4) and a central pore-like helix (M2). The S1–M1 polypeptide segment leads from the LBD in an extended conformation until it reaches the TMD. The M4 helix is located outside the ion channel domain and is linked to the LBD’s S2 segment through two helix twists and a briefly extended polypeptide region [22].

Currently, evidence indicates that several chemical groups can engage with the AMPA receptor at allosteric modulatory sites, resulting in alterations in the gating mechanism of the receptor, including processes such as activation, deactivation, and desensitization. Multiple allosteric modulators, such as 2,3-BDZ, have been extensively researched for their effects on the processes of desensitization and deactivation [9,23,24,25]. The area of 2,3-BDZ compounds has seen notable advancements, resulting in new compounds with distinct and exceptional characteristics. In addition, these substances have undergone assessment for their anxiolytic-like and antidepressant-like properties and their potential for causing memory impairment and toxicity in Swiss mice [26,27]. BDZs are a category of psychoactive substances that have been widely acknowledged for their impact on the central nervous system (CNS), namely in the regulation of GABA (γ-aminobutyric acid) and glutamate, two essential neurotransmitters responsible for maintaining brain activity equilibrium. However, GABA is the principal inhibitory neurotransmitter, and glutamate is the major excitatory complement [28]. BDZs have a facilitatory influence on the action of GABA, resulting in a net inhibitory impact on neuronal activity. Modifying physiological processes, such as drowsiness, muscular relaxation, anti-anxiety, and anticonvulsant actions, is paramount. The field of neuropharmacology emphasizes the study of 2,3-BDZs, which represent a distinct subclass within the broader category of benzodiazepines. These chemicals interact with GABA and glutamate receptors, including N-methyl-D-aspartate (NMDA) and AMPA receptors. The capacity to regulate both excitatory and inhibitory neurotransmission endows them with the capability to impact diverse neurological processes, therefore offering prospective therapeutic prospects for the treatment of numerous neurological illnesses. The distinctive capacity of 2,3-BDZs to modulate AMPA receptors sets them apart from other benzodiazepines. The selective engagement of these interactions can potentially mitigate excitotoxicity and seizure activity, presenting a potentially revolutionary treatment approach for a range of neurological illnesses that require the delicate equilibrium of excitatory and inhibitory neurotransmission.

**Figure 1:**
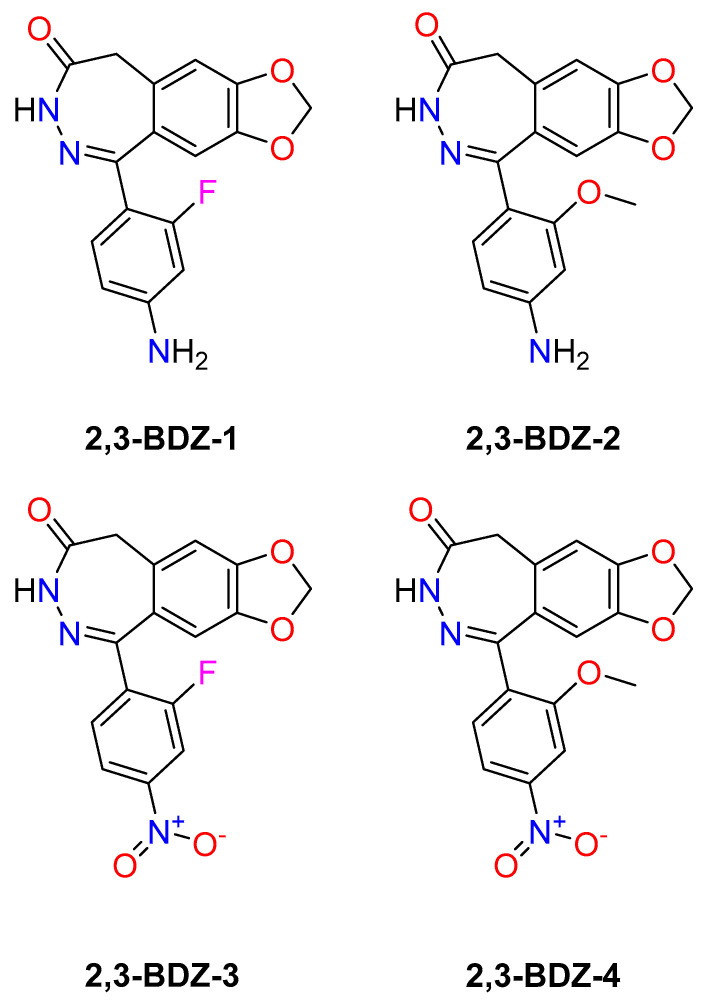
The chemical structures of the novel 2,3-BDZ derivatives.

In addition, recent advancements in understanding the composition and operation of 2,3-BDZs with AMPA receptors have laid the groundwork for creating innovative molecules that selectively interact with these receptors [29]. Early research revealed that binding sites for negative allosteric modulators (NAMs) were situated in the LBD-TMD linker region and that residues in the linkers joining the S1-M1 and S2-M4 areas affected NAM inhibitory activity [30,31]. The implications of binding to S1-M1 (Lys 506–Gly 513) and S2-M4 (Gly 774–Ser 788) linkers on gating characteristics suggest that these linkers undergo a shift during channel gating. The proposal suggests that the movement in question is a crucial step in converting agonist binding to channel opening and that the hindrance of this movement plays a pivotal role in the molecular mechanism of NAM inhibition. Upon binding to S1-M1 and S2-M4, these compounds stabilize the linker regions, thereby impeding the generation of a conformational change sufficient for the agonist contact to facilitate channel opening [22,31]. Investigating how these ligands alter channel gating may provide further information about receptor-function mechanisms. We used electrophysiological recordings to investigate how four novel 2,3-BDZ derivatives (2,3-BDZ-1-2,3-BDZ-4) (Figure 1) bind and impact AMPA receptors.

During the 1990s, many research groups started chemical and pharmacological studies to learn more about the structure-activity relationship of benzodiazepines [32], a group of compounds that selectively block AMPA receptors without competing with them, and to find new derivatives with better pharmacological properties. The GYKI 52466 prototype, belonging to the family of allosteric modulators, exhibits anticonvulsant properties across various seizure models. Despite the comprehensive exploration and lucid explication of structure-activity relationship investigations, the pharmacological characteristics of the most favorable derivatives necessitate significant enhancement [33]. GYKI-52466 and GYKI-53405 are gold standards in AMPA receptor-mediated neuronal activity research. A few hundred analogs have resulted in a thorough knowledge of replacing various side chains in the fundamental chemical structure of 2,3-BDZ [32]. Previously, our lab investigated two sets of novel 2,3-BDZ derivatives to better understand the structure-activity relationship and how modifications in the side chains may impact the compounds’ ability to inhibit the AMPAR subunits. In our initial study on 2,3-BDZ compounds, we directed our attention toward particular attributes: eliminating the amine group from the phenyl ring and introducing a halogen group at either the meta or ortho position of the phenyl ring. Our observations revealed that the presence of an amino group is not essential for inhibition when an electron-withdrawing group is positioned at the meta site. In our subsequent investigation concerning 2,3-BDZ derivatives, we examined the impact of incorporating an electron-withdrawing group, specifically Cl or Br, and the significance of the phenyl ring.

Additionally, we assessed the potential cytotoxic effects of compounds **4b** and **4e**. Our findings suggest that these additions significantly contribute to the inhibition of AMPA receptors. Furthermore, compounds **4b** and **4e** exhibited promising cytotoxicity against diverse cancer cell lines. As mentioned, the information enhances comprehension about AMPARs, presents alternative medication candidates of 2,3-BDZ distinct from standard derivatives, and potentially functions as neuroprotective agents [25,34]. However, it is uncertain how much chemical structural changes affect the selectivity of newly synthesized drugs for AMPA receptors. The research will be enhanced by investigating homomeric and heteromeric AMPA receptor subunits.

Given the intricate nature of AMPA receptor activity and the possible therapeutic implications associated with 2,3-BDZa, this work aims to examine their interconnection comprehensively. The primary aims of this study are to create new 2,3-BDZ derivatives through synthesis methods and analyze their chemical properties. Additionally, the study seeks to evaluate the effects of these newly synthesized derivatives on AMPA receptor channels using electrophysiological recordings. Furthermore, the study aims to investigate the potential of these derivatives as therapeutic agents for neurological disorders involving excitotoxicity and neurotransmission imbalances. The hypothesis in this study suggests that the new compounds of 2,3-BDZs would exhibit notable interactions with the AMPA receptors, affecting their gated characteristics. This, in turn, may provide prospective avenues for treatments for diverse neurological disorders. The primary objective of this study is to provide significant contributions to understanding the interactions between 2,3-BDZs and AMPA receptors, enhancing our knowledge of their prospective uses in neuropharmacology.

## 2. Results

### 2.1. Chemistry

**Scheme 1:**
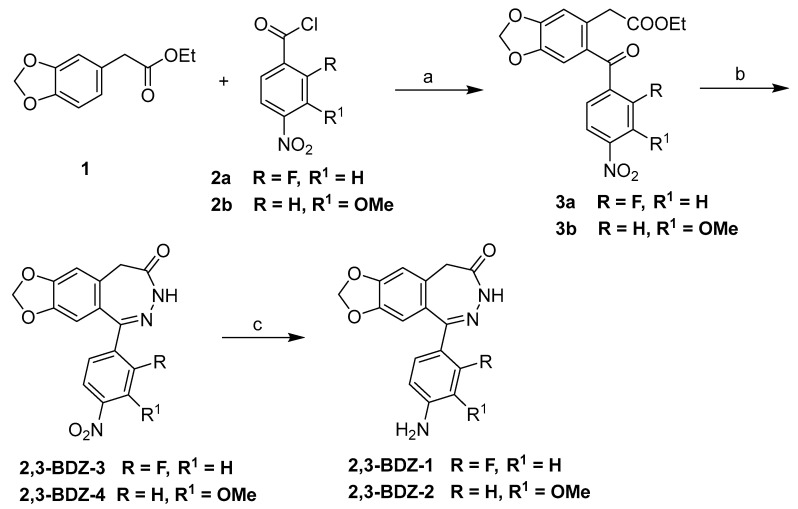
Synthesis of compound 2,3-BDZ-2. (**a**) SnCl_4_, DCM, 0 °C, then rt, overnight (**b**) NH_2_NH_2_.H_2_O, EtOH, acetic acid, reflux, 48 h (**c**) 5% Pd/C, methanol, rt, overnight.

The synthesis of target compounds is carried out according to the sequence of reactions detailed in Scheme 1. SnCl_4_-catalyzed Friedel-Craft acylation of ethyl 3,4-methylenedioxy phenylacetate 1 with either 2-fluoro-4-nitrobezoylchloride **2a** or 3-methoxy-4-nitrobenzoyl chloride **2b** in dry CH_2_Cl_2_ yields the required ketoester intermediates **3a** and **3b** in 40% and 48% yields, respectively. Ethyl 4,5-(methylenedioxy)-2-(2-fluoro-4-nitrobenzoyl) phenyl acetate **3a** was treated with hydrazine in refluxing ethanol to yield diazepin 2,3-BZD-3 in 44% yield, after which the nitro-group was reduced by Pd-catalyzed hydrogenation to yield the desired amine 2,3-BZD-1, which is found to be highly insoluble. Acetic acid promoted a cascade reaction (imine formation followed by cyclization) of ethyl 4,5-(methylenedioxy)-2-(3-methoxy-4-nitrobenzoyl) phenyl acetate **3a** with hydrazine hydrate in ethanol afforded 2,3-BZD-4 in a 33% yield. High yield and purity of 2,3-BZD-1 were achieved via Pd-catalyzed hydrogenation of the nitro group in 2,3-BZD-4 in methanol. The generated compounds have physical and spectral (1H-NMR) data consistent with their proposed structures.

### 2.2. 2,3-BDZ Compounds Have a Direct Impact on AMPA Receptor Subunits Responses

**Figure 2:**
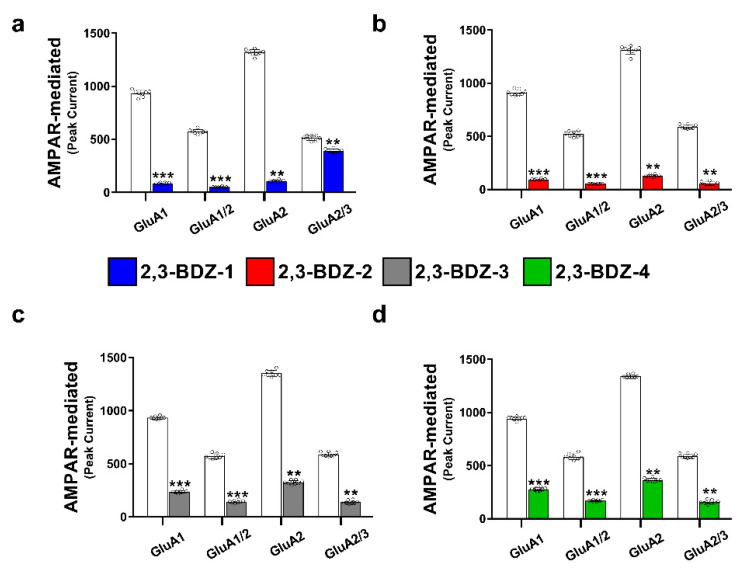
2,3-BDZ compounds affect AMPA receptor currents and synaptic responses. Figures (**a**–**d**) depict the amplitude (in picoamperes) of whole-cell recordings following the application of 10 mM glutamate alone (in white) and glutamate in combination with 2,3-BDZ-1, 2,3-BDZ-2, 2,3-BDZ-3, and 2,3-BDZ-4 Compounds (blue, red, gray, and green, respectively) at a concentration of 16 µM, as obtained from AMPAR cells expressed in HEK293T. The experiment involves recording a whole-cell current under controlled conditions of pH 7.4, temperature of 22 °C, and a voltage of −60 mV. The data are presented in the mean ± standard error of the mean (SEM), with a sample size of *n* = 8 representing the number of patch cells in the whole-cell configuration. The dots plotted above each column depict the current generated by each cell of the eight cells that were examined following the application of the compound. These dots have been graphed to visually represent the corresponding column. The statistical significance of the one-way ANOVA results is denoted as follows: (**) *p* < 0.01, and (***) *p* < 0.001.

HEK293T cells are used to clone and express AMPA receptor subunits [35], which are then recorded using the whole-cell patch-clamp technique after administering the four new 2,3-BDZ compounds. The 2,3-BDZ compounds are evaluated on four homomeric and heteromeric AMPAR subunits (i.e., GluA1, GluA1/2, GluA2, and GluA2/3) to determine their inhibitory activity on the AMPA receptor. The results show that the four 2,3-BDZ compounds have inhibitory activity against all tested subunits (Tables S1–S4, Figure 2a–d). According to Table S1, 2,3-BDZ-1 is found to have the most effective inhibitory effect on AMPA receptor subunits, with an 11–12-fold reduction in AMPAR subunit currents. While 2,3-BDZ-2 suppresses AMPAR subunit responses by approximately 10-fold (Table S2). Furthermore, AMPAR subunits are inhibited 4 and 3-fold after applying 2,3-BDZ-3 and 2,3-BDZ-4, respectively. The currents generated by applying glutamate alone (A) and glutamate plus 2,3-BDZ compounds (A_I_) are shown in Figure S1a–d. Assessing the A/A_I_ ratio enables researchers to investigate the impact of the compound on the equilibrium between excitatory and inhibitory currents facilitated by AMPA receptors. Through the analysis of the A/AI ratio, valuable insights can be obtained regarding the impact of the compound on the equilibrium between these two categories of currents. The typical excitatory current, primarily facilitated by the activation of AMPA receptors, is represented by the normal current. Conversely, the inhibitory current denotes the inhibitory neurotransmission that occurs in response to the administered compound.

### 2.3. The 2,3-BDZ Compounds Substantially Inhibited the GluA2 Subunit

**Figure 3:**
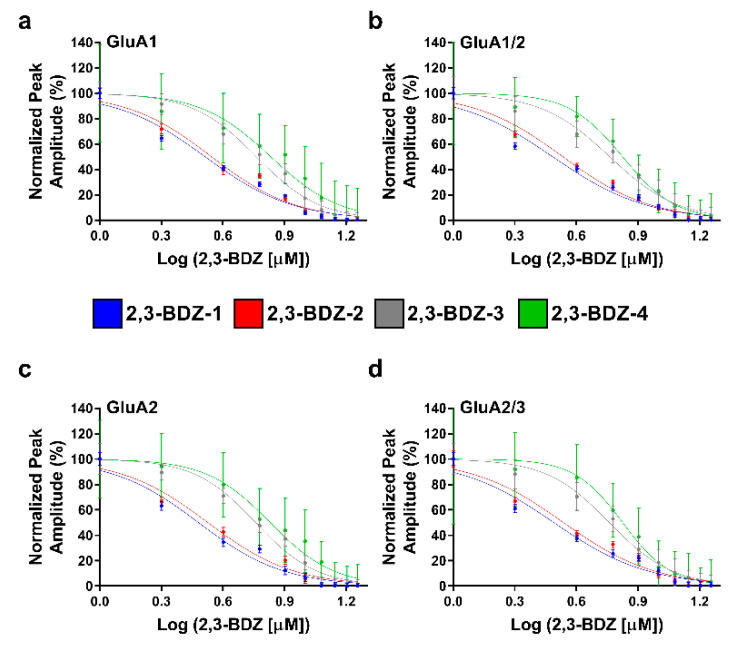
Concentration-dependent effect of 2,3-BDZ Compounds on AMPA receptor inhibition. The peak amplitudes are normalized and represented as a percentage of the control value to force the curve to run from 100% to 0%. Whole-cell responses to inhibitors are normalized to currents The normalized current amplitude derived from GluA1 subunit (**a**), GluA1/2 subunit (**b**), GluA2 (**c**), and GluA2/3 subunit (**d**) after exposure to the same set of 2,3-BDZ compounds were measured at −60 mV, pH 7.4, and 22 °C. Individual cells are exposed to different concentrations of 2,3-BDZ compounds. Each concentration is applied to the cell for 500 ms before being rinsed out for 20 ms. Following many washout periods, glutamate (10 mM) is supplied alone. Symbols/error bars represent the mean ± SEM for 8 cells.

According to the results, 2,3-BDZ compounds reduced all of the examined homomeric and heteromeric AMPAR subunit currents; however, it was noticed that the GluA2 subunit was impacted slightly more than the other four 2,3-BDZ compounds. Furthermore, the IC_50_ calculations (Table S5, Figure 3) confirm the electrophysiological recordings, which show that GluA2 is more impacted than the other tested subunits. According to Table S5, 2,3-BDZ-1 inhibits GluA2 with an IC_50_ of 3.02 µM, whereas 2,3-BDZ-2 inhibits it with an IC_50_ of 3.36 µM. Furthermore, IC_50_ values of 5.74 µM and 6.75 µM can inhibit GluA2 by 2,3-BDZ-3 and 2,3-BDZ-4, respectively. Table S5 further revealed that the examined heteromeric subunits containing GluA2 (i.e., GluA1/2 and GluA2/3) required a lower IC_50_ concentration values of each 2,3-BDZ compound to be inhibited than the GluA1 subunit. These values indicate that the compound had the greatest impact on GluA2, requiring a lower concentration to achieve the same inhibitory effect. This aligns with the understanding that a lower IC_50_ value signifies a higher effectiveness of the compound in inhibiting the target, even at relatively low concentrations. Furthermore, when examining the A/A_I_ ratios of the different subunits, GluA2 consistently exhibited the highest ratios among all the subunits tested for the four compounds, as indicated in Tables S1–S4. The higher A/A_I_ ratios observed for GluA2 highlight its greater susceptibility to modulation by these compounds and further underscore the potential significance of GluA2 in mediating excitatory responses in the context of this study.

### 2.4. 2,3-BDZ-1 and 2,3-BDZ-2 Altered AMPA Receptor Channel-Gating Kinetics

**Figure 4:**
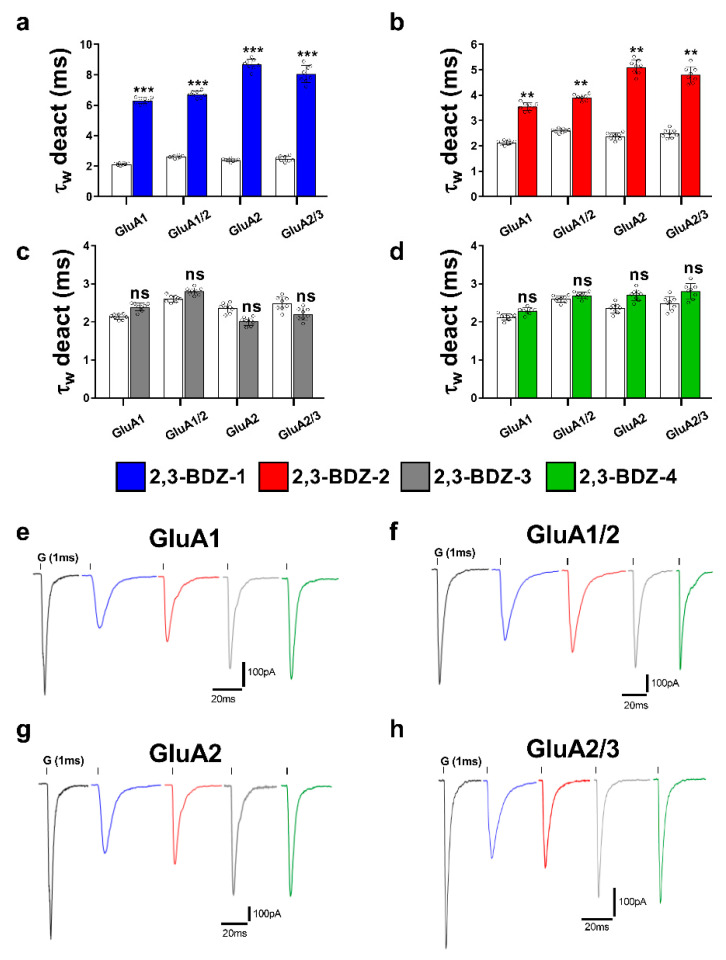
Deactivation rate (τ_w_ deact) of AMPAR subunits after applying the 2,3-BDZ derivatives. The normalized whole-cell current traces of AMPAR were recorded upon 1 ms application of 10 mM glutamate (G, indicated above the current trace) to whole-cell recording from HEK293T cells expressing homomeric GluA1 (**e**), GluA2 (**g**), heteromeric GluA1/2 (**f**) and heteromeric GluA2/3 (**h**), with glutamate alone (black) or in combination with 2,3-BDZ-1 (16 µM) (Blue), 2,3-BDZ-2 (16 µM) (Red), 2,3-BDZ-3 (16 µM) (Gray), or 2,3-BDZ-4 (16 µM) (Green)—the Mean Weighted Tau Desensitization presented in Figures (**a**–**d**). The whole-cell current is recorded under controlled conditions of pH 7.4, temperature of 22 °C, and a voltage of −60 mV. The data are presented in the format of mean ± SEM, with a sample size of *n* = 8 patch cells in the whole-cell configuration. The dots plotted above each column depict the current generated by each cell of the eight cells that were examined following the application of the compound. These dots have been graphed to visually represent the corresponding column. The statistical significance of the one-way ANOVA was determined using the following criteria: (**) *p* < 0.01, (***) *p* < 0.001, and ns indicating not significant.

**Figure 5:**
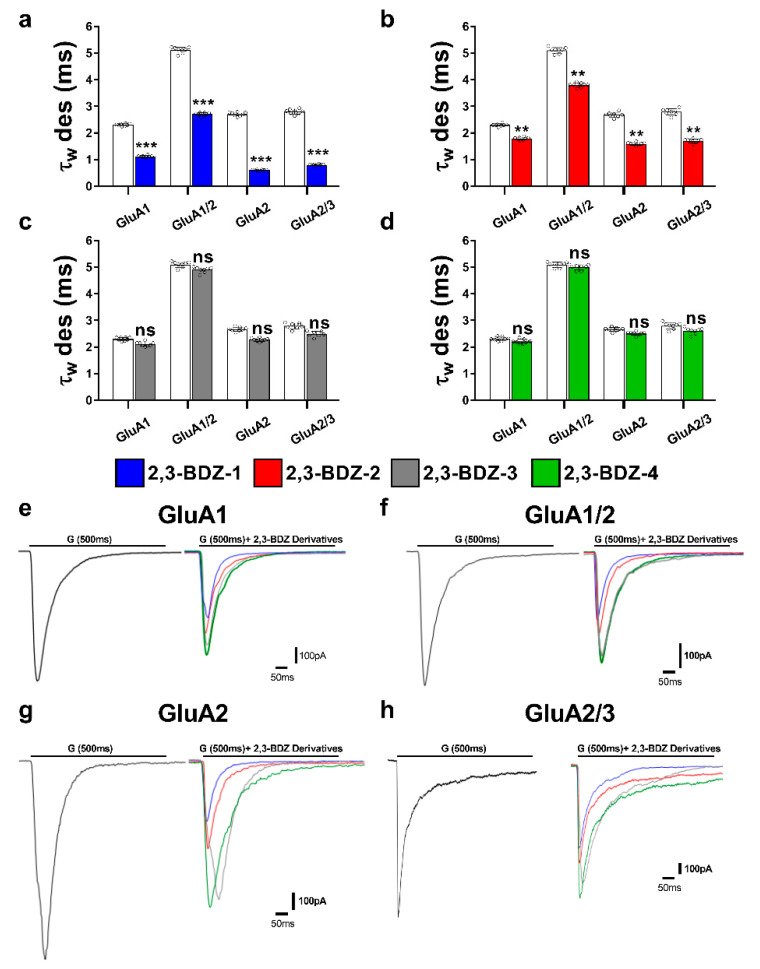
Desensitization rate (τ_w_ des) of AMPAR subunits after applying the 2,3-BDZ derivatives. The normalized whole-cell current traces of AMPAR were recorded in HEK293T cells expressing homomeric GluA1 (**e**), GluA2 (**g**), heteromeric GluA1/2 (**f**), and heteromeric GluA2/3 (**h**). The application of 10 mM glutamate, indicated above the current trace as G, was carried out for 500 ms. The traces depict the response of glutamate alone (black) or in combination with 2,3-BDZ-1 (16 µM) (Blue), 2,3-BDZ-2 (16 µM) (Red), 2,3-BDZ-3 (16 µM) (Gray), and 2,3-BDZ-4 (16 µM) (Green)—the Mean Weighted Tau Desensitization presented in Figures (**a**–**d**). The whole-cell current is recorded under controlled conditions of pH 7.4, temperature of 22 °C, and a voltage of −60 mV. The data are presented as mean ± SEM, with a sample size of *n* = 8 patch cells in the whole-cell configuration. The dots plotted above each column depict the current generated by each cell of the eight cells that were examined following the application of the compound. These dots have been graphed to visually represent the corresponding column. The statistical significance of the one-way ANOVA was determined using the following criteria: (**) *p* < 0.01, (***) *p* < 0.001, and ns denoting not significant.

The 2,3-BDZ compounds are tested to determine how they influence the kinetic aspects of AMPA receptors (such as desensitization and deactivation). According to our findings, each of the four compounds specifically affects AMPAR gating properties. For example, 2,3-BDZ-1 almost triples the rate of deactivation of the AMPAR subunits examined (Figure 4a), while 2,3-BDZ-2 nearly doubles the deactivation rate of the tested subunits (Figure 4b). Figure 4e–h display the recorded traces depicting the response of the four tested subunits when exposed to a 1 ms application of glutamate and the tested compounds. On the other hand, 2,3-BDZ-1 reduces the desensitization rate of GluA1 and GluA1/2 by almost 2-fold (Figure 5a), while it reduces the desensitization rate of GluA2/3 by almost 3–4-fold (Figure 5a). In contrast, the desensitization rate of GluA2 was reduced by a-fold 4–5 when 2,3-BDZ-1 was applied (Figure 5a). In addition, 2,3-BDZ-2 causes a 1–2-fold reduction in the desensitization rate of the investigated subunits (Figure 5b). Surprisingly, neither 2,3-BDZ-3 nor 2,3-BDZ-4 affect the desensitization or deactivation rates (Figure 4c,d and Figure 5c,d). Figure 5e–h display the recorded traces depicting the response of the four tested subunits when exposed to a 500 ms application of glutamate and the tested compounds.

## 3. Discussion

The primary objective of this research endeavor was to investigate the impact of four novel derivatives of 2,3-BDZs, namely 2,3-BDZ-1 through 2,3-BDZ-4, on specific components of the AMPA receptor. This work aimed to investigate the inhibitory effects of these compounds and their rates of desensitization and deactivation. The main result of our investigation is that the 2,3-BDZ compounds have inhibitory characteristics on both homomeric and heteromeric AMPAR subunits. All four compounds exhibited inhibitory effects on AMPAR currents, with 2,3-BDZ-1 and 2,3-BDZ-2 displaying the highest effectiveness in suppressing AMPAR activity. The results of this research highlight the potential of these chemicals as prospective candidates for the modification of AMPA receptor activity.

Furthermore, it was noted that the 2,3-BDZ compounds had a greater effect on the GluA2 subunit compared to the other subunits. Both 2,3-BDZ-1 and 2,3-BDZ-2 had an increased inhibitory impact on the GluA2 subunit, indicating that GluA2 potentially assumes a pivotal role as a target for the mechanism of action of these drugs. The results of our study indicate that the effective manipulation of 2,3-BDZ compounds in modulating the GluA2 subunit can significantly impact the regulation of AMPA receptors. The comprehension of the relative potencies of diverse derivatives on distinct AMPAR subunits is particularly significant in clarifying the structural and functional variances among these subunits. It offers a valuable understanding of synaptic plasticity and excitotoxicity mechanisms. The post-transcriptional editing process occurs at a specific site known as the Q/R site of GluA2, with a precise location at position 607. The M2 segment is observed to extend into the pore, resulting in the non-rectifying nature of the channel and its impermeability to calcium. This highlights the crucial involvement of GluA2 in the mechanism, as mentioned earlier [36].

Different AMPAR subunits have distinct structural and functional features, and targeting various subunits allows for more extensive AMPAR activity control. In the case of chronic pain, targeting both GluA2/3 and GluA1/2 has been more successful than targeting GluA2 solely in lowering pain sensation and avoiding the development of central sensitization [37]. Targeting GluA2-containing AMPARs alone may not be sufficient to avoid excitotoxicity and neuronal injury while targeting additional AMPAR subunits such as GluA2/3 complexes is more beneficial [38]. It is noteworthy because 2,3-BDZ compounds might operate on GluA2 homomers, reducing their unusual conductance property when desensitized [39].

The inhibitory effects of 2,3-BDZ-1 and 2,3-BDZ-2 are substantially stronger than those of 2,3-BDZ-3 and 2,3-BDZ-4, according to the current study results. The structural difference between the first two compounds (2,3-BDZ-1 and 2,3-BDZ-2) and the final two compounds (2,3-BDZ-3 and 2,3-BDZ-4) is that the nitro group in 2,3-BDZ-1 and 2,3-BDZ-2 is reduced to an amino group positioned on the phenyl ring in the para position. While the nitro group works as a hydrogen bond acceptor, the ability of the amino group to establish hydrogen bonds with the carbonyl groups of the receptor may explain the difference in inhibitory action between the two sets of molecules. Moreover, the amino group at the para position of the phenyl ring may bind to the amino acids in the AMPA receptor structure more strongly than the nitro group.

Furthermore, 2,3-BDZ-1 inhibited AMPA receptor subunits the most significantly. One probable reason is that the amino group of the phenyl ring is in the para position, while an electron-drawing group (fluorine) is in the ortho position. For the reason that fluorine has the greatest electronegativity of any element, its group has the largest electron-withdrawing potential. Fluorine-containing organic compounds have been shown to alter intermolecular interactions in ligand-protein complexes and to create hydrogen bonds with the receptor. Furthermore, fluorine has the greatest affinity for glycine, which is one of the amino acids found at the S1-M1 (Lys 506-Gly 513) and S2-M4 (Gly 774-Ser 788) linkers, where NAMs often bind AMPAR [40,41]. The observed inhibitory properties of 2,3-BDZ derivatives on AMPA receptor subunits can be attributed to distinct interactions between these compounds and the structural components of the receptor. Identifying functional groups such as amino and nitro groups in the 2,3-BDZ compounds indicates the possibility of establishing hydrogen bonding interactions with specific amino acid residues located within the subunits of the AMPA receptor. This interaction can potentially enhance the stability of the complex formed between the ligand and the receptor.

Moreover, the benzene rings present in the compounds have the potential to participate in π-π interactions with aromatic residues located in the receptor. This interaction mechanism significantly contributes to the binding affinity observed between the compounds and the receptor. The 2,3-BDZ compounds may experience electrostatic interactions with charged groups in the receptor’s binding pocket. These interactions can potentially stabilize the ligand and receptor complex by engaging with oppositely charged residues. Van der Waals, specifically London dispersion forces, may contribute to molecular binding.

Furthermore, there may be domain-specific interactions where various 2,3-BDZ compounds selectively target specific regions, such as the ligand-binding domain or amino-terminal domain, thereby influencing the functioning of the receptor. The specific characteristics of these interactions may exhibit variability contingent upon the subunit composition of the receptor complex. By using advanced computational methods and doing mutagenesis studies, we might be able to learn more about the specific binding mechanisms and essential amino acid residues that are key to these interactions [25,29].

Our prior investigation examined the impact of two distinct 2,3-BDZ derivatives on AMPAR subunits. Remarkably, we discovered that the presence of an amino group on the phenyl ring was not essential for inhibition. Instead, we found that an electron-drawing group could effectively replace the amino group in mediating inhibition. Moreover, our findings demonstrated that 2,3-BDZs containing an electron-withdrawing group in the meta position exhibited greater efficacy in inhibiting AMPAR than those with an electron-withdrawing group in the ortho position [34]. Following this, we conducted subsequent experiments involving five additional 2,3-BDZ derivatives. These experiments’ results confirmed that adding an electron-withdrawing group, such as Cl or Br, to the phenyl ring significantly enhanced the compounds’ ability to inhibit AMPAR currents [25]. These additional findings further support the structural requirements for AMPAR inhibition by 2,3-BDZ derivatives and highlight the potential significance of electron-withdrawing groups in improving their inhibitory effects. Such insights could be valuable in advancing future drug development efforts related to AMPAR modulation. Replacing the amino group with electron-drawing groups in 2,3-BDZ derivatives may lead to enhanced inhibition of AMPA receptors through specific mechanisms. Firstly, these electron-drawing groups can augment the phenyl ring’s electron-withdrawing ability, thereby increasing the compound’s propensity to interact with the receptor’s active site more effectively.

Additionally, introducing electron-drawing groups can enhance the compound’s lipophilicity, making it more likely to traverse the blood-brain barrier and reach the brain tissue, facilitating interactions with AMPA receptors at the target site. However, while these explanations offer potential reasons for the observed effects, a comprehensive understanding of the underlying mechanisms requires further investigation. Detailed studies elucidating the specific interactions between the compounds and the receptor’s binding site will provide valuable insights into the structure-activity relationship and the potential therapeutic applications of these novel 2,3-BDZ derivatives.

The diverse range of chemical compounds in the 2,3-BDZ family has attracted significant interest, specifically tofisopam (commercially known as Grandaxin), girisopam, and nerisopam. Tofisopam exhibits distinctive anxiolytic properties that set it apart from conventional benzodiazepines. It interacts with specific AMPA receptor sites instead of the GABA receptors that sedative benzodiazepines typically target, giving it this distinct advantage. Furthermore, the anxiolytic effects of girisopam, while not yet fully understood, may potentially involve the modulation of AMPA receptors. This suggests the possibility of exploring new therapeutic approaches for anxiety disorders. Although less extensively studied, Nerisopam presents an additional prospective contender for AMPA receptor interaction, owing to its chemical lineage within the 2,3-BDZ family. Within the framework of our research, which extensively examined the effects of 2,3-BDZ derivatives such as 2,3-BDZ-1 to 2,3-BDZ-4 on AMPARs, a coherent and persuasive narrative arises regarding the wider scope of the 2,3-BDZ family in modulating these receptors. In particular, a comprehensive analysis of the structural distinctions among these compounds, compared to tofisopam and similar substances, could yield valuable insights into the therapeutic subtleties observed within this group. This thorough investigation has the potential to enhance our current understanding, providing valuable insights for future studies and advancements in the treatment of neurological disorders associated with excessive AMPA receptor activation [27,42].

The present study offers valuable strengths and insights into the effects of 2,3-BDZ compounds on AMPA receptor subunits, encompassing both homomeric and heteromeric subunits. This investigation sheds light on the inhibitory mechanisms of these compounds on AMPA receptors, providing potential avenues for developing effective treatments for neurological disorders associated with AMPA receptor overstimulation. Notably, the study identifies that 2,3-BDZ-1 and 2,3-BDZ-2 uniquely affect the desensitization rates of AMPARs, representing a novel discovery for an AMPAR inhibitor.

However, future research must address certain limitations to interpret the findings comprehensively. Firstly, the specific binding sites of the 2,3-BDZs on the AMPAR subunits remain limited, necessitating further research to understand the precise interaction between these compounds and the receptor. Secondly, the mechanisms by which the 2,3-BDZs alter the deactivation and desensitization rates of AMPARs are yet to be fully elucidated, requiring further investigation to unravel the underlying molecular processes. Several additional variables, such as voltage dependence, ionic flux, and experimental conditions, must be meticulously regulated to ensure the accuracy of electrophysiological data. While the study’s use of recombinant AMPA receptor subunits expressed in human embryonic kidney cells enables precise electrophysiological recordings and exploration of specific subunit combinations, it fails to replicate the brain’s cellular environment’s complexity fully. Future research should investigate the impact of 2,3-BDZs on AMPA receptor complexes in an in vivo setting to understand their effects comprehensively.

Moreover, addressing the constraints of in vitro experimentation and exploring the potential clinical uses of 2,3-BDZs as AMPA receptor antagonists for neurological disorders associated with excessive receptor stimulation are essential for future research endeavors. Independent replication of the study is crucial to validating the observed effects and establishing the robustness of the findings. To further our understanding, exploring the specific binding sites and interactions between 2,3-BDZ compounds and AMPA receptor subunits is imperative, offering deeper insights into the molecular mechanisms of inhibition. Additionally, investigating the broader physiological consequences of these compounds in neurological disorders and synaptic function is vital to assess potential off-target effects and overall impacts on neuronal excitability, synaptic plasticity, and cognitive function in vivo. By addressing these limitations, future research can effectively advance the therapeutic potential of 2,3-BDZ compounds in treating neurological disorders.

The 2,3-BDZ family holds substantial therapeutic potential and promising applications in treating neurological disorders. The results of this study lay the groundwork for future research and refinement of 2,3-BDZ derivatives to enhance their specificity, effectiveness, and safety characteristics. By refining the chemical structure, scientists can develop compounds with enhanced specificity towards specific subunits of the AMPA receptor, enabling precise interventions within specific brain regions or circuits. Additionally, investigating how 2,3-BDZ and other allosteric modulators work together may lead to new combinations that synergistically improve therapeutic outcomes. Due to the many ways AMPA receptors are involved in synaptic plasticity, memory consolidation, and excitotoxicity, more research is needed to see if 2,3-BDZ could treat neurological disorders caused by abnormal AMPA receptor activity. These compounds are promising therapeutic agents in conditions such as epilepsy, traumatic brain injury, and stroke, where abnormal AMPA receptor activity contributes to neuronal damage.

Moreover, it is critical to explore their potential as analgesics for managing chronic pain, especially in cases with central sensitization and heightened pain perception. Advancements in drug delivery technologies present an opportunity to develop precise delivery systems for 2,3-BDZ. Such systems can enhance brain penetration while minimizing adverse effects on peripheral tissues, optimizing the therapeutic range, and reducing potential adverse reactions. Safety considerations are paramount, and thorough assessments and preclinical research are essential to uncovering any side effects and long-term consequences of prolonged use. Understanding the impact of 2,3-BDZ on other neurotransmitter systems and brain functions is vital to avoiding unintended effects. Translational research that leads to clinical trials is needed to determine if 2,3-BDZ derivatives are safe and effective for people with neurological disorders. Implementing a methodical approach that considers patient subgroups and individual variability is crucial to identifying positive responders and optimizing treatment strategies. Despite the therapeutic potential of these compounds, several important considerations need resolution. Achieving desired levels of selectivity is a significant challenge due to the widespread distribution of AMPA receptors in the brain and their involvement in various physiological processes. Selectively targeting specific subunits may reduce unintended effects and enhance therapeutic efficacy. Furthermore, it is important to figure out how 2,3-BDZ affects synaptic plasticity, learning, and memory since these receptors are so important to these functions. Striking a balance between inhibition and excitotoxicity is essential to preserving proper brain function and preventing detrimental consequences. To optimize treatment outcomes, the permeability of these compounds across the blood-brain barrier (BBB) needs consideration. Precise drug delivery to the brain while minimizing systemic exposure and peripheral side effects is critical. Long-term safety testing is important because long-term use of AMPA receptor antagonists may cause neuroadaptations that change how receptors work, synapses work, and neural circuits work. Diligent patient monitoring is essential for detecting adverse effects and assessing the likelihood of tolerance development. Finally, acknowledging individual variability in response, including genetic variations and comorbidities, underscores the importance of personalized treatment strategies in clinical settings. Addressing these considerations will advance the therapeutic potential of 2,3-BDZ, paving the way for more effective and targeted interventions in neurological disorders [43,44,45].

## 4. Materials and Methods

### 4.1. Chemistry

The NMR spectra were acquired utilizing a Varian Gemini 2000 instrument (1H 300 MHz, 13C 75.45 MHz) or a Bruker Avance 300 (1H 300 MHz, 13C 75.45 MHz) spectrometer. Chemical shifts (δ) in ppm are given relative to Me4Si coupling constants (J) in Hz. EI mass spectra were recorded with a Micromass GC-T. Melting points were recorded with an electrothermal melting point apparatus and were uncorrected. Analytical TLC was carried out on Merck 5785 Kieselgel 60F254 fluorescent plates. Flash chromatography uses silica gel of 35–70 µm particle size. Dimethylformamide was distilled from magnesium sulfate. Phosphorus pentoxide, SnCl4, and 2-Fluoro-4-nitrobenzoic acid were purchased from Sigma-Aldrich (Darmstadt, Germany). Dichloromethane, diethyl ether, and hexane-ethyl acetate were procured from Sigma-Aldrich (Darmstadt, Germany).

#### 4.1.1. Ethyl 4,5-(Methylenedioxy)-2-(2-fluoro-4-nitrobenzoyl) Phenyl Acetate **3a**

2-Fluoro-4-nitrobenzoyl chloride 2a (2.7 g, 14.6 mmol) and SnCl4 (7.0 g, 26.9 mmol) were sequentially added to a solution of ethyl 3,4-(methylenedioxy)-phenylacetate 1 (2.0 g, 9.6 mmol) in DCM (50 mL). The experiment was conducted under ambient conditions in the presence of nitrogen gas. The concoction was agitated under ambient conditions and subsequently thinned down with frigid water at a gradual pace. The mixture was carefully neutralized with solid K_2_CO_3_ before being extracted with 1:1 hexane/EtOAc (2 × 300 mL). The extracted mixture was washed with water and brine, dried over MgSO_4_, and filtered. The solvent was eliminated via reduced pressure, and the resultant residue underwent purification through column chromatography using (hexane/EtOAc 3:1) to yield the title compound **3a** as a yellow solid (1.73 g, 48%); 1H NMR (CDCl3) δ ppm: 1.25 (t, *J* = 7.1 Hz, 3H, CH_3_), 3.95 (s, 2H, CH2-CO), 4.16 (q, *J* = 7.1 Hz, 2H, OC-O-CH_2_), 6.05 (s, 2H, O-CH_2_-O), 6.81 (d, *J* = 1.6 Hz, 1H, ArH), 6.83 (s, 1H, ArH), 7,72 (dd, *J* = 8.5, 6.7 Hz, 1H, ArH), 8.02 (dd, *J* = 8.5, 2.1 Hz, 1H, ArH), 8.11 (ddd, *J* = 8.9, 2.1, 0.7 Hz, 1H, ArH); 13C NMR (CDCl3) δ ppm: 14.2, 40.0, 61.0, 102.4, 111.6, 112.3, 113.0, 119.4, 129.8, 131.7, 132.4, 133.6, 147.0, 151.4, 158.2, 160.7, 171.2, 191.0.

##### 7,8-(Ethylenedioxy)-1-(2-fluoro-4-nitrophenyl)-3,5-dihydro-4H-2,3-benzodiazepin-4-one (2,3-BDZ-3)

A solution of ethyl 4,5-(methylenedioxy)-2-(2-fluoro-4-nitrobenzoyl) phenyl acetate **3a** (1 g, 2.67 mmol) and hydrazine hydrate (166 µL, 3.38 mmol) in ethanol (30 mL) was refluxed for 4 days. The mixture was subjected to cooling at ambient temperature, followed by solvent evaporation under reduced pressure. The resultant yellow solid was recrystallized from methanol, yielding the requisite 2,3-BDZ-3 (0.41 g, 44%); mp 182–184 °C. IR: 1655 cm^−1^ amide carbonyl (C=O) stretching, 1514.50 cm^−1^ asymmetric nitro (N-O) stretching, 3324.91 cm^−1^ (N-H) amide stretching, 1H NMR (DMSO-d6) δ ppm: 3.42 (s, 2H, -CH2-CO-), 6.10 (s, 2H, O-CH2-O), 7.00 (s, 1H), 7.04 (s, 1H), 7.85 (dd, *J* = 8.9, 0.7 Hz, 1H, ArH), 7.95 (dd, *J* = 8.9, 2.0 Hz, 1H), 8.50 (dd, *J* = 2.0, 0.7 Hz, 1H), 8.96 (br s, 1H, NH); 13C NMR (CDCldelta ppm 38.0, 101.9, 108.0, 110.4, 111.0, 115.5, 122.4, 124.9, 125.3, 129.5, 146.4, 149.6, 147.9, 170.2.

##### 1-(4-Amino-2-fluorophenyl)-7,8-(methylenedioxy)-3,5-dihydro-4H-2,3-benzodiazepin-4-one (2,3-BDZ-1)

5% Pd/C (50 mg) was added to a solution containing 1-(2-floro-4-nitrophenyl)-7,8-(methylenedioxy)-3,5-dihydro-4H-2,3-benzodiazepin-4-one (2,3-BDZ-3) (64.80 mg, 0.19 mmol) in methanol (5 mL) and formic acid (2 mL). After 48 h of stirring at ambient temperature in a hydrogen atmosphere, the Pd/C was filtered out, and the solvent was removed under pressure. The resultant residue was washed with methanol and then with diethyl ether. The resulting residue was washed with methanol and then diethyl ether. The product 2,3-BDZ-1 was obtained as a green solid (56 mg, 94.92%): decomposition occurred at 330 °C; IR: 3122.96 cm^−1^ (N-H) stretching, 1681.92 cm^−1^ (C=O) stretching for amide, 1336.66 cm^−1^ (C-N) stretching, 1H NMR (DMSO-d6) δ We were unable to conduct NMR due to the compound’s low solubility.

#### 4.1.2. Ethyl 2-(2-Methoxy-4-nitrobenzoyl)-4,5-(methylenedioxy) Phenylacetate **3b**

To a solution of ethyl 3,4-(methylenedioxy) phenylacetate 1 (1.18 g, 5.67 mmol) in CH2Cl2 (5 mL), 2-methoxy-4-nitrobenzoyl chloride 2b (1.63 g, 7.6 mmol), and SnCl4 (1.18 mL, 10.36 mmol) were added sequentially at 0 C under a nitrogen atmosphere. Upon gradually increasing the temperature of the reaction mixture to ambient conditions. After 42 h of agitation at room temperature, the mixture was poured into saturated NaHCO_3_ (40 mL) and extracted with ethyl acetate (3 ×60 mL). The amalgamated extract of ethyl acetate underwent a process of aqueous and brine washing, followed by desiccation using MgSO_4_. The solvent was subjected to evaporation under reduced pressure. Subsequently, the resulting residue underwent purification via column chromatography (7.5:2.5 hexane/EtOAc) to furnish the title compound **3b** as a thick oil (0.88 g, 40%). IR: 1731.36 (C=O) cm^−1^ ester, 1662.03 cm^−1^ (C=O) ketone, 1521.81 cm^−1^ (N-O) stretching; 1H NMR (DMSO-d6) δ 1.16 (t, *J* = 7.1 Hz, 3H), 3.84 (s, 3H), 3.90 (s, 2H), 4.05 (t, *J* = 7.1 Hz, 2H), 6.12 (s, 2H), 6.81 (s, 1H), 7.06 (s, 1H), 7.47 (d, *J* = 8.2 Hz, 1H), 7.89 (d, *J* = 2.2 Hz, 1H), 7.91 (dd, *J* = 8.2, 2.2 Hz, 1H); 13C NMR (CDCldelta ppm 14.6, 40.5, 57.0, 60.6, 102.9, 107.6, 113.3, 113.7, 116.3, 129.9, 132.9, 136.1, 146.6, 149.9, 151.3, 157.4, 171.5, 194.1.

##### 1-(2-Methoxy-4-nitrophenyl)-7,8-(methylenedioxy)-3,5-dihydro-4H-2,3-benzodiazepin-4-one (2,3-BDZ-4)

A solution of ethyl 2-(2-methoxy-4-nitrobenzoyl)-4,5-(methylenedioxy) phenylacetate **3b** (0.72 g, 1.86 mmol), hydrazine hydrate (556 μL, 1.16 mmol), and acetic acid (50 μL) in ethanol (15 mL) was agitated at reflux for 8 days. The reaction mixture was cooled, the solvent was withdrawn under reduced pressure, and the resultant yellow solid was purified by recrystallization from ethyl acetate to provide the desired product 2,3-BDZ-4 (0.22 mg, 33%) as a pale yellow solid. 1H NMR (DMSO-d6) δ 2.08 (s, 2H), 3.72 (s, 3H), 6.05 (s, 2H), 6.40 (s, 1H), 7.07 (s, 1H), 7.70 (d, *J* = 8.3 Hz, 1H), 7.83 (d, *J* = 2.2 Hz, 1H), 7.95 (dd, *J* = 8.3, 2.2 Hz, 1H). 13C NMR (CDCl3) δ ppm: 31.3, 41.5, 57.0, 102.5, 116.2, 126.1, 130.5, 131.6, 134.8, 146.9, 149.5, 150.5, 156.5, 158.1, 159.9, 169.2.

##### 1-(2-Methoxy-4-aminophenyl)-7,8-(methylenedioxy)-3,5-dihydro-4H-2,3-benzodiazepin-4-one (2,3-BDZ-2)

5% Pd/C (0.03 g) was added to a suspension of 2,3-BDZ-4 (0.12 g, 0.33 mmol) in methanol (5 mL). The concoction was agitated in a hydrogen environment for 18 h. Using celite as a filter, Pd/C was removed before the solvent was evaporated at low pressure, yielding a light brown solid that, after being washed with ethyl acetate and diethyl ether, yielded the required amine 2,3-BDZ-2 as a greenish solid (0.052 g, 47%); 1H NMR (DMSO-d6) δ 2.08 (s, 2H), 3.41 (s, 3H), 5.45 (s, 1H), 6.03 (s, 2H), 6.20 (s, 1H), 6.21 (dd, *J* = 8.1, 2.11H), 7.70 (d, *J* = 8.3 Hz, 1H), 7.83 (d, *J* = 2.2 Hz, 1H), 7.95 (dd, *J* = 8.3, 2.2 Hz, 1H), 7.09 (d, *J* = 8.0 Hz, 1H); 13C NMR (CDCl3) δ ppm: 31.2, 41.6, 55.7, 97.7, 102.2, 106.3, 107.4, 116.1, 128.2, 130.0, 131.3, 146.4, 150.0, 152.2, 158.9, 160.1, 170.5.

### 4.2. Whole-Cell Patch-Clamp Electrophysiology

For the DNA preparation, the QIAGEN Plasmid Mini Kit was used to prepare up to 20 µg of high-copy plasmid DNA [30]. All AMPAR subunits utilized in this study included the flip isoform received from S. F. Heinemann (Salk Institute, La Jolla, CA, USA) and sub-cloned in pRK for expression in Human Embryonic Kidney Cells 293 (HEK293T). The human embryonic kidney (HEK293T) cells were obtained from Sigma, Taufkirchen, Germany, and we only used the HEK293T cell line due to their ease of growth and transfection and wide utilization in cell culture. For the transfection, we used the constructs GluA1, GluA2, GluA2/3, and GluA1/2, along with the GFP-expressing construct, as described in our previous work. The transfection reagents utilized were jetPRIME (Polyplus: New York, NY, USA) or Lipofectamine 2000 (Invitrogen; San Diego, CA, USA). The highly fluorescent cells were identified and selected for recording. The selected cells were seeded in Dulbecco Modified Eagle Medium (DMEM) (Sigma, Roedermark, Germany) containing 10% FBS, 0.1 mg/mL streptomycin, and 1 mM sodium pyruvate (Biological Industries; Beit-Haemek, Israel). The cells were incubated at 37 °C with 5% CO_2_ added to the medium and subcultured twice a week until they reached passage #20. Following a day and a half, the cells were replated on coverslips covered with Laminin (1 mg/mL; Sigma, Roedermark, Germany) for electrophysiological recordings. Our previous work has detailed the DNA preparation, cDNA transient transfection, and cell culture of HEK293T [30,31,32,33].

After that, a patch was placed in front of a rapid perfusion system comprised of a theta tube flow pipe mounted on a piezoelectric translator that was used to wash the cell continuously from one barrel while the other supplied the compound of interest to the cell. Recordings were conducted 36–48 h after transfection at 22 °C, with a membrane potential of −60 mV. Currents were amplified using an integrated patch amplifier (IPA, Sutter Instruments, Novato, CA, USA), filtered at 2 kHz, and digitized at 10 kHz using SutterPatch Software v. 1.1.1 [46]. The pipettes utilized in the experiment exhibited a resistance range of 2–4 MΩ and were loaded with a solution comprising millimolar concentrations of the following substances: 110 CsF, 30 CsCl, 4 NaCl, 0.5 CaCl_2_, 10 trypsin-EDTA solution B (0.25%), EDTA (0.05%), and 10 HEPES, pH 7.2 adjusted with CsOH. The extracellular medium contained (in mM): 150 NaCl, 2.8 KCl, 0.5 MgCl_2_, 2 CaCl_2_, and 10 HEPES, pH 7.4 adjusted with NaOH. The holding potential was typically −60 mV. The sample size was determined to be eight viable cells to calculate the mean inhibition of the derivative of interest. The solution exchange time (10–90% to peak) was estimated at 500 milliseconds (ms) using open-tip control at the end of a recording. Inhibition was calculated by comparing the current observed when the cell was supplied with glutamate alone and when the same cell was supplied with both glutamate and the 2,3-BDZ compounds. The initial tube containing only glutamate was reintroduced to the cell to ensure cellular safety and authenticate the antagonist’s findings. This step ensured that the glutamate-induced current observed before the administration of the antagonist was nearly indistinguishable from the current observed after administration.

The experiment served two purposes: first, determining the whole-cell current amplitude ratio in the absence and presence of the compounds (A/A_I_) to derive a constant inhibitory value independent of the rate measurement. Second, as an independent control method, a fast exchange solution flow methodology was employed using piezoelectric actuators, providing the fastest exchange solution with open-tip current rise times in the 100–300 μs range to assess the amplitude of the whole-cell current. Detailed data analysis of this process is provided in the supporting information (Tables S1–S4).

The data acquired were analyzed using Igor Pro7 (Wave Metrics, Inc., Portland, OR, USA). Receptor desensitization (τ_w_ des) and deactivation rates were estimated by fitting the current decay starting from 95% of the peak to the baseline current using a double exponential formulation. The double exponential model used two-time constants, τrise for the rising phase and τdecay for the decay phase of the current. While the decay phase of the current is not always well described using a single exponential, a double exponential is often used to fit the decay phase, and a weighted mean time constant (τ_w_) is extracted.

Using the weighted mean time constant (τ_w_) instead of the single decay time constant is an improved approach. Therefore, an approach based on the use of both the fast (τf) and slow (τs) time constants (τf and τs) is employed. The currents were evoked by applying 10 mM glutamate for 500 ms for desensitization and 1 ms for deactivation. The weighted tau (τ_w_) was calculated as τ_w_ = (τf x af) + (τs x as), where af and as are the relative amplitudes of the fast (τf) and slow (τs) exponential components.

The statistical analyses were executed utilizing GraphPad Prism version 6.01 (GraphPad Software). The statistical method of ANOVA was employed to assess the presence of significant variations among the four chemical compounds depicted in the figures. The level of statistical significance was established at a threshold of *p* < 0.05, denoting (*) *p* < 0.05, (**) *p* < 0.01, (***) *p* < 0.001, or ns, indicating a lack of statistical significance. Each 2,3-BDZ compound was tested on eight individual cells, with each experimental trial replicated three times. The figures presented in this study display the mean values, accompanied by the standard error of the mean (SEM).

## 5. Conclusions

AMPA receptors, classified as ionotropic glutamate receptors, facilitate rapid neurotransmission, mostly inside the central nervous system. AMPA receptors are pivotal in regulating brain activities, including synaptic plasticity, memory consolidation, and learning processes. Moreover, there is a significant association between them and the initiation of pathological conditions, such as neurodegenerative illnesses. The present investigation used four recently developed 2,3-BDZ and performed electrophysiological evaluations of AMPAR subunit currents. The results of our study suggest that these substances have inhibitory characteristics on the AMPAR channel. The findings of our investigation indicate that 2,3-BDZ derivatives with an amino group in the para position have superior inhibitory effects when compared to their nitro-substituted equivalents. Furthermore, a fluorine atom is being introduced in the ortho position of the 2,3-BDZ-1 molecule, leading to significant inhibition of both homomeric and heteromeric subunits of AMPA receptors. Both 2,3-BDZ-1 and 2,3-BDZ-2 influence the rates of deactivation and desensitization of AMPAR. Additional research is required to better understand the pharmacological properties of 2,3-BDZ and their potential therapeutic use in neurological diseases.

## Data Availability

This published article and its supplementary file include all data generated or analyzed during this study.

## Supplementary Materials

The following supporting information can be downloaded at: https://www.mdpi.com/article/10.3390/molecules28166067/s1, Figure S1: The inhibition assays of four 2,3-BDZ derivatives on GluA1, GluA2, GluA1/2, and GluA2/3 subunits; Table S1: Whole-cell recordings for compound 2,3-BDZ-1; Table S2 Whole-cell recordings for compound 2,3-BDZ-2; Table S3: Whole-cell recordings for compound 2,3-BDZ-3; Table S4: Whole-cell recordings for compound 2,3-BDZ-4; Table S5: IC50 values

## Sample Availability

Samples of the 2,3-BDZ compounds tested are available from the authors upon request.

## List of Abbreviations

AMPA, α-amino-3-hydroxy-5-methyl-4-isoxazole propionic acid; AMPARs, α-amino-3-hydroxy-5-methyl-4-isoxazole propionic acid receptors; HEK293T, Human embryonic kidney 293 cells; 2,3-BDZ, 2,3-benzodiazepine; CNS, central nervous system; LBD, ligand-binding domain; TMD, transmembrane domain; NAM, negative allosteric modulators.
