# Supplementary material for: Electrophysiological Assessment of Newly Synthesized 2,3-Benzodiazepine Derivatives for Inhibiting the AMPA Receptor Channel
Source: Molecules. 2023 Aug 15;28(16):6067. doi: 10.3390/molecules28166067 (PMC10458471; doi:10.3390/molecules28166067)

Article

Electrophysiological Assessment of Newly Synthesized 2,3-Benzodiazepine Derivatives for Inhibiting the AMPA Receptor Channel

Mohammad Qneibi [1,*], Hanan Jumaa [2], Sosana Bdir [1] and Nawaf Al-Maharik [2,*]

[1] Department of Biomedical Sciences, Faculty of Medicine and Health Sciences, An-Najah National
University, Nablus P.O. Box 7, Palestine; s12027767@stu.najah.edu

[2] Department of Chemistry, Faculty of Sciences, An-Najah National University, Nablus P.O. Box 7, Palestine; hjumaa97@gmail.com

* Correspondence: mqneibi@najah.edu (M.Q.); n.maharik@najah.edu (N.A.-M.)

Supplementary Material

Table S1-S4: Whole-cell recording of data analysis for 2,3-BDZ compounds;

Table S5: IC_{50} values. IC_{50} calculated values for 2,3-BDZ compounds;

Figure S1: The inhibition assays of four 2,3-BDZ derivatives on GluA1, GluA2, GluA1/2, and GluA2/3 subunits

Table of Contents

Supplemental Table S1 Whole-cell recordings for compound 2,3-BDZ-1………………..…4

Supplemental Table S2 Whole-cell recordings for compound 2,3-BDZ-2………………..….5

Supplemental Table S3 Whole-cell recordings for compound 2,3-BDZ-3……………….….….6

Supplemental Table S4 Whole-cell recordings for compound 2,3-BDZ-4…………………...7

Supplemental Table S5 IC_{50} values……………………………………………………..9

Supplemental Figure S1 The inhibition assays of four 2,3-BDZ derivatives on GluA1, GluA2, GluA1/2, and GluA2/3 subunits…………………………………………………….....10

Supplemental Table S1 Whole-Cell Recordings for Compound 2,3-BDZ-1

Receptor Name/Compounds abbreviation	GluA1 (Glutamate Alone)	2,3-BDZ-1	Applying Glutamate Alone After 2,3-BDZ-1	n	A/A_I
Amplitude (pA)	933±30	85±7.0***	935±39	8	11.08±1.0
t deact (ms)	2.1±0.1	6.3±0.4***	N/R	8	N/R
t des (ms)	2.3±0.1	1.1±0.1***	N/R	8	N/R
**Receptor Name/Compounds abbreviation**	**GluA1/2 (Glutamate Alone)**	**2,3-BDZ-1**	**Applying Glutamate Alone After 2,3-BDZ-1**	**n**	**A/A_I**
Amplitude (pA)	573±34	51±7.0***	575±35	8	11.21±1.0
t deact (ms)	2.6±0.2	6.7±0.6***	N/R	8	N/R
t des (ms)	5.1±0.4	2.7±0.2***	N/R	8	N/R
**Receptor Name/Compounds abbreviation**	**GluA2 (Glutamate Alone)**	**2,3-BDZ-1**	**Applying Glutamate Alone After 2,3-BDZ-1**	**n**	**A/A_I**
Amplitude (pA)	1318±28	108±9.0***	1322±33	8	12.31±1.0
t deact (ms)	2.4±0.1	8.7±0.6***	N/R	8	N/R
t des (ms)	2.7±0.1	0.6±0.05***	N/R	8	N/R
**Receptor Name/Compounds abbreviation**	**GluA2/3 (Glutamate Alone)**	**2,3-BDZ-1**	**Applying Glutamate Alone After 2,3-BDZ-1**	**n**	**A/A_I**
Amplitude	588±19	49±4***	589±33	8	12.09±1.0

**(pA)**					
**t deact (ms)**	2.5±0.2	8.1±0.7***	N/R	8	N/R
**t des (ms)**	2.8±0.3	0.8±0.08***	N/R	8	N/R

(***) $p < 0.001$

Supplemental Table S2 Whole-Cell Recordings for Compound 2,3-BDZ-2

**Receptor Name/Compounds abbreviation**	**GluA1 (Glutamate Alone)**	**2,3-BDZ-2**	**Applying Glutamate Alone After 2,3-BDZ-2**	**n**	**A/A$_I$**
**Amplitude (pA)**	913±37	93±10.0***	914±40	8	9.82±0.7
**t deact (ms)**	N/R	3.6±0.3**	N/R	8	N/R
**t des (ms)**	N/R	1.8±0.1**	N/R	8	N/R
**Receptor Name/Compounds abbreviation**	**GluA1/2 (Glutamate Alone)**	**2,3-BDZ-2**	**Applying Glutamate Alone After 2,3-BDZ-2**	**n**	**A/A$_I$**
**Amplitude (pA)**	521±25	53±3.0***	523±33	8	9.91±0.6
**t deact (ms)**	N/R	3.9±0.4**	N/R	8	N/R
**t des (ms)**	N/R	3.8±0.3**	N/R	8	N/R
**Receptor Name/Compounds abbreviation**	**GluA2 (Glutamate Alone)**	**2,3-BDZ-2**	**Applying Glutamate Alone After 2,3-BDZ-2**	**n**	**A/A$_I$**
**Amplitude (pA)**	1312±38	127±12.0***	1310±42	8	10.42±0.8
**t deact (ms)**	N/R	5.1±0.4**	N/R	8	N/R
**t des**	N/R	1.6±0.05**	N/R	8	N/R

**(ms)**					
**Receptor Name/Compounds abbreviation**	**GluA2/3 (Glutamate Alone)**	**2,3-BDZ-2**	**Applying Glutamate Alone After 2,3-BDZ-2**	**n**	**A/A$_I$**
**Amplitude (pA)**	536±22	52±7.0***	532±26	8	10.28±0.6
**t deact (ms)**	N/R	4.8±0.5**	N/R	8	N/R
**t des (ms)**	N/R	1.7±0.08**	N/R	8	N/R

(**) $p < 0.01$, and (***) $p < 0.001$

Supplemental Table S3 Whole-Cell Recordings for Compound 2,3-BDZ-3

**Receptor Name/Compounds abbreviation**	**GluA1 (Glutamate Alone)**	**2,3-BDZ-3**	**Applying Glutamate Alone After 2,3-BDZ-3**	**n**	**A/A$_I$**
**Amplitude (pA)**	934±13	237±12**	936±46	8	3.94±0.2
**t deact (ms)**	N/R	2.4±0.1^{ns}	N/R	8	N/R
**t des (ms)**	N/R	2.1±0.1^{ns}	N/R	8	N/R
**Receptor Name/Compounds abbreviation**	**GluA1/2 (Glutamate Alone)**	**2,3-BDZ-3**	**Applying Glutamate Alone After 2,3-BDZ-3**	**n**	**A/A$_I$**
**Amplitude (pA)**	572±24	144±11**	569±43	8	3.98±0.2
**t deact (ms)**	N/R	2.8±0.2^{ns}	N/R	8	N/R
**t des (ms)**	N/R	4.9±0.3^{ns}	N/R	8	N/R

Receptor Name/Compounds abbreviation	GluA2 (Glutamate Alone)	2,3-BDZ-3	Applying Glutamate Alone After 2,3-BDZ-3	n	A/AI
Amplitude (pA)	1352±28	326±17**	1251±65	8	4.16±0.2
t deact (ms)	N/R	2.8±0.1ns	N/R	8	N/R
t des (ms)	N/R	2.3±0.1ns	N/R	8	N/R
**Receptor Name/Compounds abbreviation**	**GluA2/3 (Glutamate Alone)**	**2,3-BDZ-3**	**Applying Glutamate Alone After 2,3-BDZ-3**	**n**	**A/AI**
Amplitude (pA)	529±12	130±12**	525±22	8	4.08±0.3
t deact (ms)	N/R	2.9±0.2ns	N/R	8	N/R
t des (ms)	N/R	2.5±0.2ns	N/R	8	N/R

(**) $p < 0.01$, and ns denoting not significant

Supplemental Table S4 Whole-Cell Recordings for Compound 2,3-BDZ-4

Receptor Name/Compounds abbreviation	GluA1 (Glutamate Alone)	2,3-BDZ-4	Applying Glutamate Alone After 2,3-BDZ-4	n	A/AI
Amplitude (pA)	944±16	277±14**	942±44	8	3.41±0.2
t deact (ms)	N/R	2.3±0.1ns	N/R	8	N/R
t des (ms)	N/R	2.2±0.1ns	N/R	8	N/R
**Receptor Name/Compounds abbreviation**	**GluA1/2 (Glutamate Alone)**	**2,3-BDZ-4**	**Applying Glutamate Alone After 2,3-BDZ-4**	**n**	**A/AI**

**Amplitude (pA)**	584±28	170±9**	583±40	8	3.44±0.2
**t deact (ms)**	N/R	2.7±0.2ns	N/R	8	N/R
**t des (ms)**	N/R	5.0±0.3ns	N/R	8	N/R
**Receptor Name/Compounds abbreviation**	**GluA2 (Glutamate Alone)**	**2,3-BDZ-4**	**Applying Glutamate Alone After 2,3-BDZ-4**	**n**	**A/AI**
**Amplitude (pA)**	1341±15	364±17**	1340±52	8	3.68±0.1
**t deact (ms)**	N/R	2.7±0.1ns	N/R	8	N/R
**t des (ms)**	N/R	2.5±0.1ns	N/R	8	N/R
**Receptor Name/Compounds abbreviation**	**GluA2/3 (Glutamate Alone)**	**2,3-BDZ-4**	**Applying Glutamate Alone After 2,3-BDZ-4**	**n**	**A/AI**
**Amplitude (pA)**	518±12	146±14**	516±23	8	3.56±0.3
**t deact (ms)**	N/R	2.8±0.2ns	N/R	8	N/R
**t des (ms)**	N/R	2.6±0.2ns	N/R	8	N/R

(**) $p < 0.01$ and ns denoting not significant

Supplemental Table S5 IC_{50} values

**Receptor/Subunit**	**2,3-BDZ Derivatives**	2,3-BDZ-1	2,3-BDZ-2	2,3-BDZ-3	2,3-BDZ-4
**GluA1**	**IC_{50}**	3.26	3.51	5.80	6.80
	**Log IC_{50}**	0.51	0.54	0.76	0.83
	**R square**	0.97	0.98	0.98	0.95

Receptor/Subunit	**2,3-BDZ Derivatives**	2,3-BDZ-1	2,3-BDZ-2	2,3-BDZ-3	2,3-BDZ-4
**GluA1/2**	**IC_{50}**	3.05	3.43	5.79	6.69
	**Log IC_{50}**	0.48	0.53	0.76	0.83
	**R square**	0.97	0.98	0.98	0.99

Receptor/Subunit	**2,3-BDZ Derivatives**	2,3-BDZ-1	2,3-BDZ-2	2,3-BDZ-3	2,3-BDZ-4
**GluA2**	**IC_{50}**	3.02	3.36	5.74	6.75
	**Log IC_{50}**	0.48	0.53	0.76	0.83
	**R square**	0.97	0.97	0.98	0.97

Receptor/Subunit	**2,3-BDZ Derivatives**	2,3-BDZ-1	2,3-BDZ-2	2,3-BDZ-3	2,3-BDZ-4
**GluA2/3**	**IC_{50}**	3.09	3.45	5.76	6.69
	**Log IC_{50}**	0.49	0.54	0.76	0.83
	**R square**	0.97	0.97	0.99	0.99

Supplemental Figure S1: The inhibition assays of four 2,3-BDZ derivatives on GluA1, GluA2, GluA1/2, and GluA2/3 subunits. These four figures depict the A/A_I ratio, which signifies the ratio between normal (excitatory) and inhibitory currents. a shows the A/A_I measured for GluA1 subunit after applying the four 2,3-BDZ compunds, while b shows the A/A_I measured for GluA1/2 subunit after applying the four 2,3-BDZ compunds, c shows the A/A_I measured for GluA2 subunit after applying the four 2,3-BDZ compunds, and d shows the A/A_I measured for GluA2/3 subunit after applying the four 2,3-BDZ compunds. The experiment involves conducting a whole-cell current recording under controlled con-ditions of pH 7.4, temperature of 22 °C, and a voltage of -60 mV. The data are presented in the format of mean ± standard error of the mean (SEM), with a sample size of n = 8 repre-senting the number of patch cells in the whole-cell configuration. The statistical signifi-cance of the one-way ANOVA results is denoted as follows: (**) $p < 0.01$, and (***) $p < 0.001$.

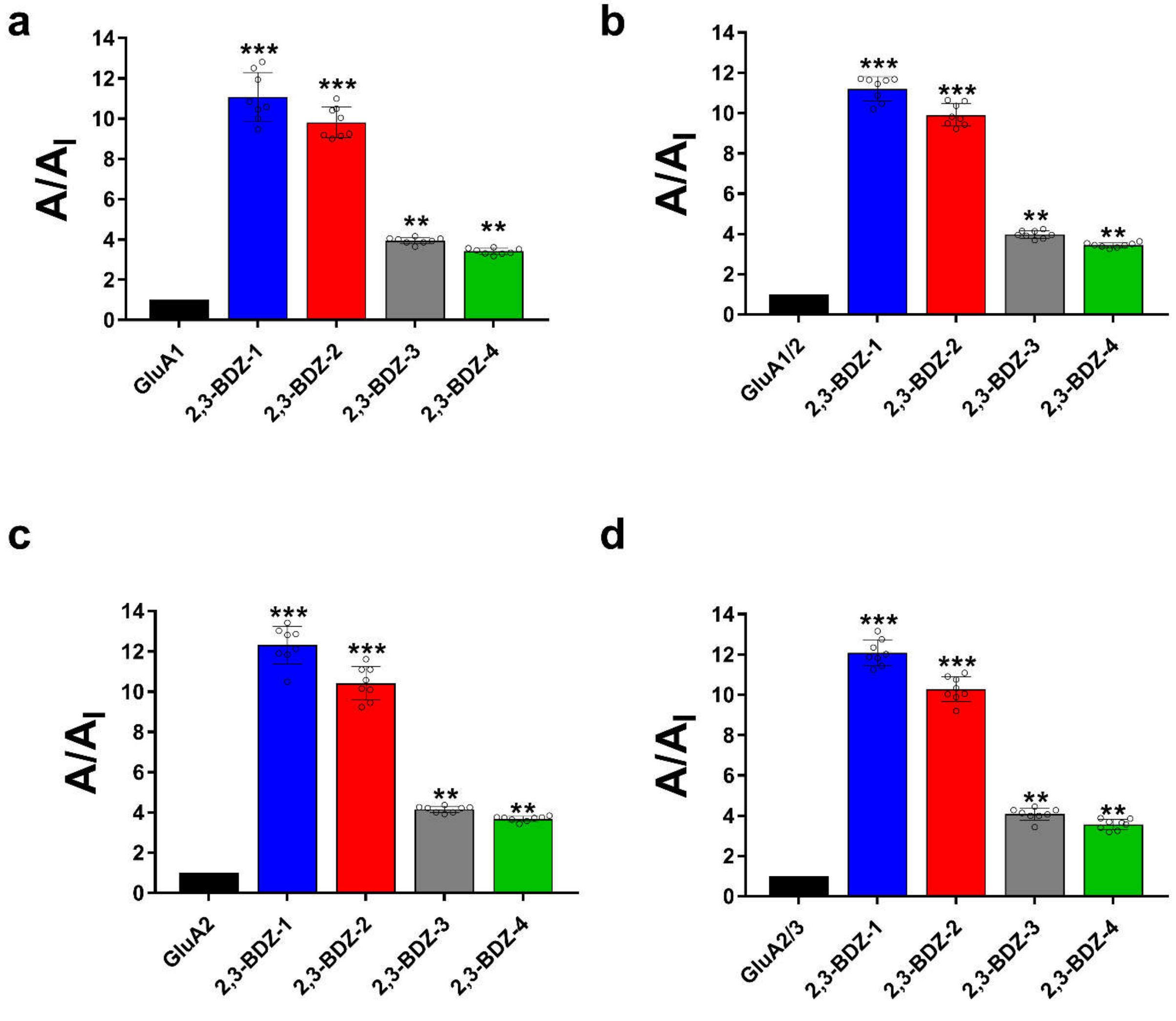

Supplement: Supplementary file 1 [file molecules-28-06067-s001.zip › molecules-2541521-supplementary.pdf]
